# A systematic review evaluating the psychometric properties of measures of social inclusion

**DOI:** 10.1371/journal.pone.0179109

**Published:** 2017-06-09

**Authors:** Reinie Cordier, Ben Milbourn, Robyn Martin, Angus Buchanan, Donna Chung, Renée Speyer

**Affiliations:** 1 School of Occupational Therapy and Social Work, Faculty of Health Sciences, Curtin University, Perth, Western Australia, Australia; 2 College of Healthcare Sciences, James Cook University, Townsville, Queensland, Australia; Western Sydney University, AUSTRALIA

## Abstract

**Introduction:**

Improving social inclusion opportunities for population health has been identified as a priority area for international policy. There is a need to comprehensively examine and evaluate the quality of psychometric properties of measures of social inclusion that are used to guide social policy and outcomes.

**Objective:**

To conduct a systematic review of the literature on all current measures of social inclusion for any population group, to evaluate the quality of the psychometric properties of identified measures, and to evaluate if they capture the construct of social inclusion.

**Methods:**

A systematic search was performed using five electronic databases: CINAHL, PsycINFO, Embase, ERIC and Pubmed and grey literature were sourced to identify measures of social inclusion. The psychometric properties of the social inclusion measures were evaluated against the COSMIN taxonomy of measurement properties using pre-set psychometric criteria.

**Results:**

Of the 109 measures identified, twenty-five measures, involving twenty-five studies and one manual met the inclusion criteria. The overall quality of the reviewed measures was variable, with the Social and Community Opportunities Profile-Short, Social Connectedness Scale and the Social Inclusion Scale demonstrating the strongest evidence for sound psychometric quality. The most common domain included in the measures was connectedness (21), followed by participation (19); the domain of citizenship was covered by the least number of measures (10). No single instrument measured all aspects within the three domains of social inclusion. Of the measures with sound psychometric evidence, the Social and Community Opportunities Profile-Short captured the construct of social inclusion best.

**Conclusions:**

The overall quality of the psychometric properties demonstrate that the current suite of available instruments for the measurement of social inclusion are promising but need further refinement. There is a need for a universal working definition of social inclusion as an overarching construct for ongoing research in the area of the psychometric properties of social inclusion instruments.

## Introduction

The concepts of social inclusion and exclusion focus on health, social, cultural and income inequalities and imbalances [1]. The term social inclusion is used in social policy and practice documents to highlight the importance of engagement and participation in society as a means of improving quality of life and reducing social isolation [2]. This is because communities that actively include and support individuals and groups to participate in valued social, economic and cultural activities are likely to be healthier than those where people face insecurity, exclusion and deprivation [3]. In order to further develop evidence about the ways in which forms of social inclusion can impact on the wellbeing of individuals, families and communities, it is important to accurately measure and report on what constitutes social inclusion. This paper sets out to consider how the concept of social inclusion has been deployed in policy and practice, how the construct has been operationalised as measures, and identifies the quality of the psychometric properties underpinning the evidence base. This will enable policy makers and practitioners to take a more evidence based approach to evaluating social inclusion initiatives in the future.

### Social inclusion: History and definition of the concept

The term social inclusion has been used variously in international social policy and academia; indicating an underpinning policy and practice intent. There is debate about what defines social inclusion, largely due to differences in theoretical and political perspectives. It has also been used interchangeably at times with concepts such as social and cultural capital. Due to this lack of consensus on definition and conceptualisation, the operationalisation and measurement of social inclusion has not been straightforward.

In order to examine the emergence of social inclusion, an inspection of the theories, policies and practices which underpin both inclusion and exclusion is required. While social exclusion and inclusion are often framed as binary opposites, some would suggest the concepts are relative and intertwined and it is not possible to understand or measure social inclusion without reference to social exclusion [4]. However, some argue the two concepts represent entirely different experiences as exclusion suggests “social problems” and inclusion indicates “social membership” [5].

The construct of social exclusion emerged from René Lenoir’s [5] ideas in the 1970s. Lenoir, a French Secretary of State, highlighted incidents of exclusion resulting from poverty, disability, substance misuse, incarceration and mental health problems [5]. Durkheimian [6] ideas about social cohesion and order underpinned the original conceptualisation of social exclusion. Identifying and addressing social exclusion then became a key social policy focus across Europe in the 1980s, Britain in the 1990s and Australia in the 2000s. The policy shift to the language of social exclusion replaced discourses of poverty and disadvantage underpinning arguments that individual welfare payments failed to address the root causes of social exclusion [7]. While the link between social exclusion and poverty has been both emphasised and minimised, some argue that poverty, unemployment and social exclusion are “related, but should not be equated” [8] (p. v), suggesting that social exclusion is a complex set of intersecting variables and experiences which cannot be attributed to one event or factor. Further, Atkinson argues that social exclusion hinges on three key concepts: a) the ‘relativity’ of spatial, temporal and cultural contexts; b) the enactment of ‘agency’ (for example he argues that some ‘choose’ unemployment); and c) the ‘dynamics’ which highlight intergenerational patterns of exclusion [8] (p. 13–14). Social exclusion and inclusion frameworks also consider citizenship and participation in paid labour [9]; which signifies a change from the original focus of exclusion of particular groups to an emphasis on economic participation [4].

Social exclusion can exist across multiple domains and often disrupts activities such as citizenship, participation, social relationships and connections, health, employment, and housing [5, 8]. Aligned with these ideas, Levitas [6] identified three discursive trends within the social exclusion literature which cover impoverishment and exclusion in the economic, social and cultural spheres; the emphasis on paid work as a form of social integration, and a focus on the specific characteristics of excluded individuals. While it is possible to trace various discursive practices in the definitions of social exclusion, it is argued that “what lies at the heart of all processes of social exclusion, is [sic] a sense of social isolation and segregation from the formal structures and institutions of the economy, society and the state” [10]. The notion of community openness to welcome or create a space for those defined as excluded was largely overlooked [5].

### Social inclusion as policy and practice

During the 1990s, the concept of social inclusion/exclusion entered state policy discourse providing a ‘catch all’ means to incorporate diverse forms of disadvantage and inequalities. This resulted in social policies across Europe, Britain, and Australia that ambitiously sought to counter the effects of social exclusion and bolster social inclusion. For example, when New Labour was elected in Britain in 1997, social inclusion was its key social policy platform. It was intended to represent a major shift away from the traditional British welfare state with which Labour had historically been associated. The then Prime Minister, Tony Blair, was personally committed to the approach and was highly influenced by the writings of prominent academic Antony Giddens who argued the aspirations for the modern welfare state had not come to fruition and there was a need for a ‘Third Way’ that did not solely rely on national level policy to reduce inequalities in Britain [11].

While the theory and conceptualisation of social exclusion and inclusion has been broad and somewhat fragmented, policy has largely been concerned with reducing unemployment and decreasing homelessness as key pathways to social inclusion. This indicates that policy has been oriented towards economic participation as the primary method for individuals to attain social inclusion. Social inclusion policies have largely promoted individual responsibility for change. However, policy implementation generally paid little attention to differences in material conditions amongst citizens who were unemployed and homeless, such as their cultural background, health, social and cultural capital. Similarly, policy has not deeply engaged with the dynamics, relativity or agency that authors like Atkinson [8] argue is associated with social exclusion. Consequently, measurement of social inclusion within the policy context has focused on the attainment or retention of employment and changes to homelessness status amongst specific populations. However, these measures alone are not adequate indicators of the levels and extent of social inclusion. In short, social inclusion has largely represented an aspirational goal, due to its relatively narrow policy operationalisation.

In contrast, social inclusion in professional practice (for example Community Development, Social Work, Public Health, and Occupational Therapy) has tended to focus on inclusion and participation of marginalised groups in social and community settings, as well as through forms of employment (open through to supported). However, it is rare to see social inclusion measured as a service outcome or indicator of success. As a practice principle underpinning human services, social inclusion has been associated with notions of citizenship and human rights. The assumption being, social connectedness and belonging are key to quality of life [12]. In practice, the social inclusion of service users has incorporated five interdependent aspects of human experience: the growth of relationships, choice and control, experiencing socially valued roles, sharing ordinary places, and contributions [13, 14]. The five aspects are not easily measured and while relative to individual service user’s contexts, enactment of agency and the dynamics associated with their specific lives [8], do not always take into account structural factors. The lack of consensus and clarity about social inclusion in practice has led to an absence of established methods of routinely measuring of the phenomenon to determine service delivery outcomes [15].

Despite the lack of consensus in definition and conceptualisation, the historical and contemporary literature highlights three overarching domains: 1) participation, 2) connectedness and a sense of belonging, and 3) citizenship and rights. For the purpose of this review, participation includes attendance and involvement [16] in social and community spaces and activities; engagement in the labour market and dynamics associated with exclusion, including intergenerational factors which may not be possible to overcome in order to participate. Similarly, participation cannot be accepted on face value. For example, employment as a form of participation is not always inclusion; particularly in marginal employment [8]. Connectedness relates to the actual and potential participation in social and community based activities, organisations, networks and relationships [17]. The relative exclusion of some groups is important to note, as is the potential for individuals to experience spatial connection, yet not have a sense of belonging [8, 18]. Citizenship is operationally defined as the “the link between the state and the individual that implies membership of some form of community…” [19] (p. 104) and is concerned with “a) the content of social rights and obligations; b) with the form or type of such obligations and rights; c) with the social forces that produce such practices; and finally d) with the various social arrangements whereby such benefits are distributed to different sectors of a society.” [20] (p. 3). Additionally, notions of agency, responsibility and the impact of others’ decisions is incorporated in our definition of citizenship [1, 8].

Useful measurements of social inclusion therefore need to include key measures of these three domains and their various components as discussed above. Only one study to date has attempted conduct a review of social inclusion measures [21]; however, the review was not systematic, was limited in scope, and did not use a standardised method to evaluate the quality of the psychometric properties. This systematic review will evaluate the measurements of social inclusion so that the evidence base for understanding the impact and effects of forms of social inclusion on individuals and communities will be better understood in the future and can be adapted and tested with a wider range of diverse groups.

### Study aim

This study aimed to identify all current measures of social inclusion for any population group, to evaluate the psychometric properties of these measures, and the extent to which the measures comprehensively evaluate the domains of social inclusion. The COSMIN taxonomy, terminology, and definitions of measurement properties for health-related patient-reported outcomes was used to appraise the psychometric properties of the instruments reviewed [22]. COSMIN provides a consensus on terminology surrounding psychometric properties and a checklist for evaluating the methodological quality of studies reporting on validity, reliability and responsiveness [23]. This study focused on assessing the validity and reliability of all reviewed measures. Evaluation of responsiveness would require a review of studies that have utilised the identified measures as an outcome assessment and would have increased the size of this review significantly. As such, an evaluation of the responsiveness of the reviewed instruments would warrant its own systematic review. Therefore responsiveness as a psychometric property was excluded from this study. It is expected that this systematic review will assist in informing choice when selecting an instrument for the measurement of social inclusion.

## Methods

The PRISMA statement guided the methodology and reporting of this systematic review. The PRISMA statement contains a 27 item checklist of elements considered essential for ensuring transparency in performing and reporting of systematic reviews [24]. A completed PRISMA checklist as it pertains to the current review is available (see S1 Table).

### Eligibility criteria

Published research articles or manuals assessing the psychometric properties of instruments designed to measure social inclusion in any population were considered for review. Studies selected for review did not have to adhere to a predetermined definition of social inclusion as it is not a narrowly agreed on concept. Rather, the following three domains of social inclusion from the literature were used to guide our review: 1) participation (i.e., economic, social and spiritual), 2) connectedness and a sense of belonging (i.e., having a sense of connectedness to family, friends, neighbours, broader community), and 3) citizenship and rights (i.e., political and general community engagement, demonstrating altruism, and having access to community services). To be selected for review, the overall construct evaluated by an instrument needed to reflect these domains in either children or adults. If social inclusion was evaluated by a single subscale and was not the overall construct assessed by an instrument, the instrument was excluded from the review. Only manuals or published articles written in English in the 20 years prior to the search were eligible for review. Instruments were eligible for review if their psychometric properties were published or updated in the last 20 years (i.e. after 1994), to only capture the psychometric quality of contemporary measures of social inclusion. Conference abstracts, other reviews, case reports, student dissertations and editorials were also excluded.

### Information sources

A systematic literature search was conducted using five electronic databases: CINAHL, Embase, ERIC, PsycINFO, and PubMed. Database searches were conducted between 28/07/2015 and the 08/08/2015. Search strategies used both free text words and subject headings, and comprised all journal articles up to August 2015. The database searches were conducted by one author (R.S.) due to her extensive experience in conducting systematic reviews. The databases were accessed from the libraries of Curtin University and James Cook University. The search strategy used for each database is reported in Table 1.

**Table 1:** Search terms.

Initial search: Assessment retrieval Database and Search Terms (Subject Headings and Free Text Words)
**CINAHL:** ((MH "Psychometrics") OR (MH "Measurement Issues and Assessments") OR (MH "Validity") OR (MH "Predictive Validity") OR (MH "Reliability and Validity") OR (MH "Internal Validity") OR (MH "Face Validity") OR (MH "External Validity") OR (MH "Discriminant Validity") OR (MH "Criterion-Related Validity") OR (MH "Consensual Validity") OR (MH "Concurrent Validity") OR (MH "Qualitative Validity") OR (MH "Construct Validity") OR (MH "Content Validity") OR (MH "Instrument Validation") OR (MH "Validation Studies") OR (MH "Test-Retest Reliability") OR (MH "Sensitivity and Specificity") OR (MH "Reproducibility of Results") OR (MH "Reliability") OR (MH "Intrarater Reliability") OR (MH "Interrater Reliability") OR (MH "Measurement Error") OR (MH "Bias (Research)") OR (MH "Selection Bias") OR (MH "Sampling Bias") OR (MH "Precision") OR (MH "Sample Size Determination") OR (MH "Repeated Measures") OR (Psychometric* or reliability or validit* or reproducibility or bias)) AND ((MH “Social Capital”) OR (MH “Social Isolation”) OR (MH “Social Justice”) OR (MH “Social Participation”) OR (MH “Social Responsibility”) OR (TI “social participation” OR AB “social participation” OR TI “social capital” OR AB “social capital” OR TI “community inclusion” OR AB “community inclusion” OR TI “social justice” OR AB “social justice” OR TI “social acceptance” OR AB “social acceptance” OR TI “social isolation” OR AB “social isolation” OR TI “social reinforcement” OR AB “social reinforcement” OR TI “social responsibility” OR AB “social responsibility” OR TI “social inclusion” OR AB “social inclusion” OR TI “community participation” OR AB “community participation”))
**Embase:** ((psychometry/ or validity/ or reliability/ or measurement error/ or measurement precision/ or measurement repeatability/ or error/ or statistical bias/ or test retest reliability/ or intrarater reliability/ or interrater reliability/ or accuracy/ or criterion validity/ or internal validity/ or face validity/ or external validity/ or discriminant validity/ or concurrent validity/ or qualitative validity/ or construct validity/ or content validity/) OR (Psychometric* or reliability or validit* or reproducibility or bias)) AND ((social acceptance/ or social capital/ or social discrimination/ or social exclusion/ or social isolation/ or social justice/ or social participation/ or social rejection/) OR (social participation.ti,ab. OR social capital.ti,ab. OR community inclusion.ti,ab. OR social justice.ti,ab. OR social acceptance.ti,ab. OR social isolation.ti,ab. OR social reinforcement.ti,ab. OR social responsibility.ti,ab. OR social inclusion.ti,ab. OR community participation.ti,ab.))
**ERIC:** ((DE "Psychometrics" OR DE "Validity" OR DE "Reliability" OR DE "Error of Measurement" OR DE "Bias" OR DE "Interrater Reliability" OR DE "Accuracy" OR DE "Predictive Validity" OR DE "Construct Validity" OR DE "Content Validity") OR (Psychometric* or reliability or validit* or reproducibility or bias)) AND ((DE “Social Capital” OR DE “Social discrimination” OR “DE “Social Isolation” OR DE “Social Justice” OR DE “Social Reinforcement” OR DE “Social Responsibility”) OR (TI “social participation” OR AB “social participation” OR TI “social capital” OR AB “social capital” OR TI “community inclusion” OR AB “community inclusion” OR TI “social justice” OR AB “social justice” OR TI “social acceptance” OR AB “social acceptance” OR TI “social isolation” OR AB “social isolation” OR TI “social reinforcement” OR AB “social reinforcement” OR TI “social responsibility” OR AB “social responsibility” OR TI “social inclusion” OR AB “social inclusion” OR TI “community participation” OR AB “community participation”))
**PsycINFO**: ((DE "Psychometrics" OR DE "Statistical Validity" OR DE "Test Validity" OR DE "Statistical Reliability" OR DE "Test Reliability" OR DE "Error of Measurement" OR DE "Errors" OR DE "Response Bias" OR DE "Interrater Reliability" OR DE "Repeated Measures") OR (Psychometric* or reliability or validit* or reproducibility or bias)) AND ((DE "Social Acceptance" OR DE "Social Capital" OR DE "Social Equality" OR DE "Social Isolation" OR DE "Social Justice" OR DE "Social Reinforcement" OR DE "Social Responsibility") OR (TI “social participation” OR AB “social participation” OR TI “social capital” OR AB “social capital” OR TI “community inclusion” OR AB “community inclusion” OR TI “social justice” OR AB “social justice” OR TI “social acceptance” OR AB “social acceptance” OR TI “social isolation” OR AB “social isolation” OR TI “social reinforcement” OR AB “social reinforcement” OR TI “social responsibility” OR AB “social responsibility” OR TI “social inclusion” OR AB “social inclusion” OR TI “community participation” OR AB “community participation”))
**PubMed:** (("Psychometrics"[Mesh] OR "Reproducibility of Results"[Mesh] OR "Validation Studies as Topic"[Mesh] OR "Validation Studies" [Publication Type] OR "Bias (Epidemiology)"[Mesh] OR "Observer Variation"[Mesh] OR "Selection Bias"[Mesh] OR "Diagnostic Errors"[Mesh] OR "Dimensional Measurement Accuracy"[Mesh] OR “Predictive Value of Tests"[Mesh] OR "Discriminant Analysis"[Mesh]) OR (psychometric* OR reliability OR validit* OR reproducibility OR bias)) AND (("Social Isolation"[Mesh] OR "Social Marginalization"[Mesh] OR "Social Capital"[Mesh] OR "Social Discrimination"[Mesh] OR "Social Participation"[Mesh] OR "Social Responsibility"[Mesh] OR "Social Justice"[Mesh] OR "Social Alienation"[Mesh]) OR (social participation[Title/Abstract] OR social capital[Title/Abstract] OR community inclusion[Title/Abstract] OR social justice[Title/Abstract] OR social acceptance[Title/Abstract] OR social isolation[Title/Abstract] OR social reinforcement[Title/Abstract] OR social responsibility[Title/Abstract] OR social inclusion[Title/Abstract] OR community participation[Title/Abstract]))

Grey literature was searched using Google Scholar. To be comprehensive, we also searched the websites of three major publishers of assessments in social sciences (Pearson, ACER and Western Psychological Services) to identify potential assessments not identified in earlier search strategies. A web search was also conducted using Google to identify any instruments available via alternative suppliers.

### Study selection

A scoring procedure was developed to screen abstracts for inclusion. Abstracts were scored by four independent raters on a three-point scale to determine if: a) the study involved a *measure* of social inclusion, b) the measure *assessed social inclusion or related terms* (e.g., social participation, social capital, social responsibility, community inclusion, social justice, social acceptance, social reinforcement, community participation), and c) the study reported on *psychometric data* of the measure. All abstracts were examined by two reviewers to determine the inter-rater reliability: Weighted Kappa = 0.750 (95% CI: 0.714–0.786). Abstracts that did not meet any of the criteria were immediately excluded from this study. Abstracts that met two or three of the criteria were each screened again by two raters to ensure only studies that met all three eligibility criteria were selected for full text extraction.

Three raters screened the extracted full texts to ensure instruments met the eligibility criteria. Measures were excluded if social inclusion was not the overall construct of the assessment (i.e., if only measured by one subscale) or if the assessment quantified social exclusion. Raters reviewed all full texts together to ensure 100% consensus on reviewed instruments.

### Data collection process and data extraction

Data to be extracted from the reviewed studies and manuals were guided by the Cochrane Handbook for Systematic Reviews section 7.3a [25], and the Systematic Reviews Centre for Reviews and Dissemination [26]. Comprehensive data collection forms were developed, and data were captured for the following parameters: study purpose, study population, age of the population, instrument purpose, measure type, number of subscales/forms, number of items, response option types, and domains of social inclusion measured. The COSMIN [23] was also used to capture data and to assess the methodological quality of the studies reviewed.

#### Methodological quality

The first phase of the review evaluated the methodological quality of the selected studies. This was performed using the COSMIN taxonomy of measurement properties and definitions for health-related patient-reported outcomes [22]. The COSMIN checklist [23] is a standardised tool for assessing the methodological quality of studies on measurement properties. It evaluates nine domains: internal consistency, reliability (relative measures: including test-retest reliability, inter-rater reliability and intra-rater reliability), measurement error (absolute measures), content validity (including face validity), structural validity, hypotheses testing, cross-cultural validity, and criterion validity. A definition of each psychometric property, as guided by the COSMIN statement, is provided in Table 2. Responsiveness was outside the scope of this review, and criterion validity was not evaluated due to the absence of a ‘gold standard’ measure of social inclusion. Cross-cultural validity was not evaluated as the instruments reviewed were developed and published in English, and interpretability is not considered to be a psychometric property under the COSMIN framework and was therefore not described in this review. The domains of the COSMIN checklist contain 5 to 18 items rated on a four-point scale (poor, fair, excellent, good). The items rate the quality of study design and the robustness of statistical analyses conducted in studies of reliability, validity and responsiveness.

**Table 2:** COSMIN: Definitions of domains, psychometric properties, and aspects of psychometric properties for health-related patient-reported outcomes based on Mokkink, Terwee [22].

Psychometric property	Domain and Definition^a^
	**Reliability:** the degree to which the measurement is free from measurement error.
Internal consistency	The degree of the interrelatedness among the items.
Reliability	The proportion of the total variance in the measurements which is because of “true” differences among patients.
Measurement error	The systematic and random error of a patient’s score that is not attributed to true changes in the construct to be measured.
	**Validity:** the degree to which an instrument measures the construct(s) it purports to measure.
Content validity	The degree to which the content of an instrument is an adequate reflection of the construct to be measured.
**Face validity**^**b**^	The degree to which (the items of) an instrument indeed looks as though they are an adequate reflection of the construct to be measured.
Construct validity	The degree to which the scores of an instrument are consistent with hypotheses based on the assumption that the instrument validly measures the construct to be measured.
**Structural validity**^**c**^	The degree to which the scores of an instrument are an adequate reflection of the dimensionality of the construct to be measured.
**Hypotheses testing**^**c**^	Item construct validity.
**Cross-cultural validity**^**c**^	The degree to which the performance of the items on a translated or culturally adapted instrument are an adequate reflection of the performance of the items of the original version of the instrument.
Criterion validity	The degree to which the scores of an instrument are an adequate reflection of a “gold standard”.
Responsiveness	**Responsiveness:** the ability of an HR-PRO instrument to detect change over time in the construct to be measured.
Interpretability^d^	**Interpretability**^**a**^: the degree to which one can assign qualitative meaning to an instrument’s quantitative scores/ score change.

Notes

^a^ Applies to Health-Related Patient-Reported Outcomes (HR-PRO) instruments

^b^ Aspect of content validity under the domain of validity

^c^ Aspects of construct validity under the domain of validity

^d^ Interpretability is not considered a psychometric property.

To allocate an overall methodological quality score to each study an alternative system to that which was proposed by the authors of COSMIN was utilised. Terwee, Mokkink [27] suggest taking the lowest rating of any item in a checklist domain as the final quality rating for that domain. It has been noted that subtle differences in the methodological quality between studies are difficult to detect via this method of scoring [28], so a revised scoring procedure was used in this study is as follows. Outcomes are presented as a percentage calculated using the following formula to ensure scores are not unfairly weighted by items that only provide ratings options at the extreme ends of the ordinal scale (i.e., “excellent” and “good”; “fair” and “poor”).

$$Total\;score\;for\;psychometric\;property\;=\;\frac{Total\;score\;obtained-minimum\;score\;possible}{Max\;score\;possible-minimum\;score\;possible}\times 100$$

The overall percentage calculated is then categorised as either Poor (0–25.0%), Fair (25.1%-50.0), Good (50.1%-75.0%), or Excellent (75.1%-100.0%). To ensure consistency in the ratings, the sixth author trained four independent research assistants to complete the COSMIN checklist. A random selection of 72% of psychometric property domians were rated by at least two raters. If a discrepancy in COSMIN ratings occurred between raters, articles were given the highest rating percentage if both ratings fell within the same category (i.e. poor, fair, good or excellent). Raters met until 100% consensus was reached when ratings differed in category (ICC = 0.888 [95% CI: 0.795–0.940]).

#### Quality of psychometric properties

Phase two assessed the quality of the psychometric properties measured in each study. The results of each study were evaluated using criteria set out by Terwee, Bot [29] and Schellingerhout, Verhagen [30] and Table 3 provides a summary of these criteria. Studies that received a “poor” methodological quality rating in phase one were excluded from further analysis and received a score of NE (not evaluated) in phase two. The raters from phase one also completed phase two, and a random selection of 72% of psychometric properties were evaluated by at least two raters. Raters met until 100% consensus was reached if psychometric quality ratings differed.

**Table 3:** *Revised* quality criteria for measurement properties of health status questionnaires based on Terwee, Bot [29] and Schellingerhout, Verhagen [30].

Property	Definition ^a^	Score ^b^	Quality criteria ^c^^,^ ^d^^,^ ^e^
Internal consistency	The extent to which items in a (sub) scale are inter-correlated, thus measuring the same construct	+	Factor analyses performed on adequate sample size (7 * # items and ≥ 100) AND Cronbach’s alpha(s) calculated per dimension AND Cronbach’s alpha(s) between 0.70 and 0.95
		?	No factor analysis OR doubtful design or method
		-	Cronbach’s alpha(s) <0.70 or >0.95, despite adequate design and method
		±	Conflicting results
		NR	No information found on internal consistency
		NE	Not evaluated
Reliability (inter rater reliability, intra rater reliability, repeated measurement)	The extent to which patients can be distinguished from each other, despite measurement errors (relative measurement error)	+	ICC or weighted Kappa ≥ 0.70
		?	Doubtful design or method (e.g., time interval not mentioned)
		-	ICC or weighted Kappa < 0.70, despite adequate design and method
		±	Conflicting results
		NR	No information found on reliability
		NE	Not evaluated
Measurement error	The extent to which the scores on repeated measures are close to each other (absolute measurement error)	+	MIC < SDC OR MIC outside the LOA OR convincing arguments that agreement is acceptable
		?	Doubtful design or method OR (MIC not defined AND no convincing arguments that agreement is acceptable)
		-	MIC ≥ SDC OR MIC equals or inside LOA, despite adequate design and method
		±	Conflicting results
		NR	No information found on agreement
		NE	Not evaluated
Content validity	The extent to which the domain of interest is comprehensively sampled by the items in the questionnaire	+	A clear description is provided of the measurement aim, the target population, the concepts that are being measured, and the item selection AND target population and (investigators OR experts) were involved in item selection
		?	A clear description of above-mentioned aspects is lacking OR only target population involved OR doubtful design or method
		-	No target population involvement
		±	Conflicting results
		NR	No information found on target population involvement
		NE	Not evaluated
Structural validity	The degree to which the scores of an HR-PRO instrument are an adequate reflection of the dimensionality of the construct to be measured	+	Factors should explain at least 50% of the variance
		?	Explained variance not mentioned
		-	Factors explain < 50% of the variance
		±	Conflicting results
		NR	No information found on structural validity
		NE	Not evaluated
Hypotheses testing	The extent to which scores on a particular questionnaire relate to other measures in a manner that is consistent with theoretically derived hypotheses concerning the concepts that are being measured	+	Specific hypotheses were formulated AND at least 75% of the results are in accordance with these hypotheses
		?	Doubtful design or method (e.g., no hypotheses)
		-	Less than 75% of hypotheses were confirmed, despite adequate design and methods
		±	Conflicting results between studies within the same manual
		NR	No information found on hypotheses testing
		NE	Not evaluated
Criterion validity	The extent to which scores on a particular questionnaire relate to a gold standard	+	Convincing arguments that gold standard is “gold” AND correlation with gold standard ≥0.70
		?	No convincing arguments that gold standard is “gold” OR doubtful design or method
		-	Correlation with gold standard <0.70, despite adequate design and method
		±	Conflicting results
		NR	No information found on criterion validity
		NE	Not evaluated

Notes

^a^ Scores: + = positive rating,? = Unknown rating,— = negative rating, ± = conflicting data, NR = not reported, NE = not evaluate

^b^ Doubtful design or method = lacking of a clear description of the design or methods of the study, sample size smaller than 50 subjects (should be at least 50 in every (subgroup) analysis), or any important methodological weakness in the design or execution of the study

^c^ Not evaluated = study of poor methodological quality according to COSMIN rating, data are excluded from further analyses

^d^ Measurement error: MIC = minimal important change, SDC = smallest detectable change, LOA = limits of agreement

^e^ Hypotheses testing: all correlations should be statistically significant (if not, these hypotheses are not confirmed) AND these correlations should be at least moderate (*r* > 0.5).

#### Overall quality of psychometric properties

During the third and final phase, each measurement property for all instruments was given an overall quality score using criteria set out by Schellingerhout, Verhagen [30]. These criteria combine the scores of study quality obtained in phase one with the psychometric quality ratings measured in phase two, thereby creating an overall quality rating. A description of this process is provided in Table 4. Two of the raters from phases one and two gave an overall quality score to each instrument and conferred over discrepancies until 100% consensus was reached.

**Table 4:** *Revised* levels of evidence for the overall quality of the measurement properties based on Schellingerhout, Verhagen [30].

Level	Criteria
Strong	Consistent findings in multiple studies of good methodological quality OR in one study of excellent methodological quality
Moderate	Consistent findings in multiples studies of fair methodological quality OR in one study of good methodological quality
Limited	One study of fair methodological quality
Conflicting	Conflicting findings
Not Evaluated^1^	Only studies of poor methodological rating
Indeterminate^2^	Only indeterminate data on measurement properties

Notes

^1^Not evaluated = only studies of poor methodological quality according to COSMIN

^2^Indeterminate = only indeterminate outcome data on the assessment measurement property, therefore, also indeterminate level of evidence for the overall quality of that measurement property.

### Data items, risk of bias and synthesis of results

Data items for each instrument were obtained. When an item was not reported, an ‘NR’ was recorded. Risk of bias was assessed at an individual study level during the rating of the COSMIN checklist in phase one. Studies obtaining high ratings during phase one are at low risk of bias, and studies with low ratings are at high risk of bias. Further risk for bias was assessed during phase two, as psychometric domains only received a “positive” or “negative” result if clear and appropriate methodology was reported. Any studies with unclear methodological reporting received an “indeterminate” rating as poor methodology left results open to bias. As the ratings from phase one and two were combined to create an overall rating for each psychometric property of each instrument, the risk of bias is subsumed into the final results. The results were synthesised and grouped as follows: 1) development and validation of the instrument, 2) the psychometric properties of the instruments, and 3) the instrument characteristics.

## Results

### Systematic literature search

**Fig 1:**
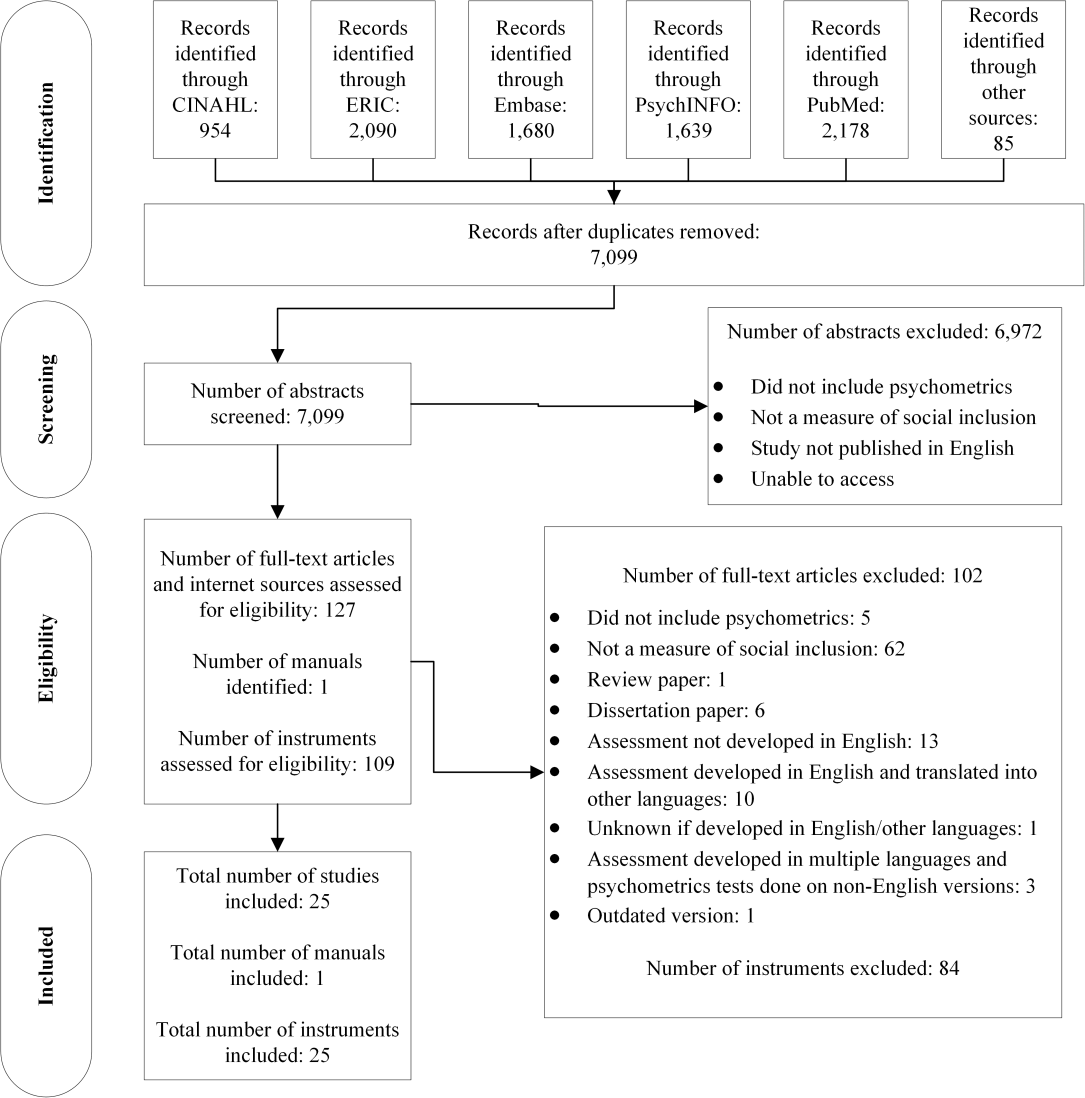
Flow diagram of the reviewing process according to PRISMA.

A total of 8,541 abstracts were retrieved from five databases with the following breakdown: CINAHL = 954, ERIC = 2,090, Embase = 1,680, PsycINFO = 1,639, PubMed = 2,178. Fig 1 presents the flow diagram of the revision process according to PRISMA [24]. Reference lists of the reviewed articles were examined for further publications meeting the eligibility criteria. The grey literature search identified an additional 85 records. A total of 1,442 duplicates across the five databases were removed, leaving a total of 7,099 studies to screen for inclusion in this review. Following abstract screening, 127 full-text articles reporting on 108 different instruments were further assessed for eligibility. Of these 108 measures, 84 were excluded for the following reasons: 1 was published before 1994, 3 did not report psychometric data, 6 were published in dissertations, 23 were developed or published in languages other than English, and 51 did not measure the domains of social inclusion adopted for this review. Table 5 lists the 84 excluded instruments and reasons for their exclusion. One manual was located through additional searches. Thus, the psychometric properties were obtained for a total of 25 social inclusion measures which were accessed using 25 articles and 1 manual.

**Table 5:** Overview of social inclusion instrument: Reasons for exclusion.

**Assessment name**	**Abbreviation**	**Reason for exclusion**
Perceived Sense of Community Scale **[31]**	N/A	Published prior to 1994
The Social Inclusion for people with Mental Illness—Long Edition **[32]**	SIMI-LE	Dissertation not published
California Health Interview Survey **[33]**	CHIS	Dissertation
Youth Outcome Questionnaire **[34]**	N/A	Dissertation
Bangladesh Social Capital Measure **[35]**	N/A	Dissertation
Bonding Social Capital Measurement Tool ^a^ **[36]**	N/A	Dissertation
Perceived Support for College Measure ^a^ **[37]**	N/A	Dissertation
Self-efficacy for social participation **[38]**	SESP	Not developed in English
Scale of Social Acceptance ^a^ **[39]**	N/A	Not developed in English
Community Commitment Scale **[40]**	CCS	Not developed in English
Social Participation Questionnaire **[41]**	N/A	Not developed in English
Maastricht Social Participation Profile **[42]**	N/A	Not developed in English
The Interview for Assessment of Social Isolation (German title: Interview zur Messung Sozialer Isolation) **[43]**	IMSI	Not developed in English
The Institute for Social Research instrument for social exclusion ^a^ **[44]**	N/A	Not developed in English
Netherlands Social Capital Index **[45]**	N/A	Not developed in English
16-item Perceived Group Inclusion Scale **[46]**	PGIS	Not developed in English
18 item Lubben Social Network Scale to Mongolian **[47]**	LSNS-18-M	Developed in English then translated into other languages
Short version of the Adapted Social Capital Assessment Tool **[48]**	SASCAT	Developed in English then translated into other languages
The Youth Capital scale **[49]**	YSCS	Developed in English then translated into other languages
The Greek version of the Social Capital Questionnaire **[50]**	SCQ-G	Developed in English then translated into other languages
The Korean Version of the Personal and Social Responsibility Questionnaire **[51]**	PSRQ	Developed in English then translated into other languages
Impact on Participation and Autonomy Scale for people with SPI **[52]**	N/A	Developed in English then translated into other languages
Persian version of Social Capital Questionnaire **[53]**	P-SCQ	Developed in English then translated into other languages
Jessor and Jessor Social Alienation Scale **[54]**	N/A	Developed in English then translated into other languages
Perceived Community Support Questionnaire **[55]**	PCSQ	Unknown if developed in English/other languages
The Participation Scale **[56]**	P-scale	Developed in multiple languages
The Participation Scale Short **[56]**	PSS	Developed in multiple languages
Personal Social Capital Scales **[57]**	PSCS-8	Developed in multiple languages
Personal Social Capital Scales 16 **[57]**	PSCS-16	Developed in multiple languages
Social capital scale ^a^ **[58]**	N/A	No psychometric data found
Open Hearts **[59]**	N/A	No psychometric data found
Everybody Active **[60]**	N/A	No psychometric data found
The Social Wellbeing Scale **[61]**	SWBS	Not a measure of Social Inclusion
ICF–Mental–A&P **[62]**	N/A	Not a measure of Social Inclusion
Bonding and Bridging Social Capital Development ^a^ **[63]**	N/A	Not a measure of Social Inclusion
The Perceived Adolescent Relationship Scale **[64]**	N/A	Not a measure of Social Inclusion
Neurologic Quality of Life **[65]**	NeuroQOL	Not a measure of Social Inclusion
The Social Participation Scale **[66]**	N/A	Not a measure of Social Inclusion
The Social Profile **[67]**	N/A	Not a measure of Social Inclusion
The National Social Life, Health and Aging Project measure ^a^ **[68]**	N/A	Not a measure of Social Inclusion
Peer Affiliation and Social Acceptance **[69]**	PASA	Not a measure of Social Inclusion
Craig Hospital Inventory of Environmental Factors **[70, 71]**	CHIEF	Not a measure of Social Inclusion
MND SOCIAL Withdrawal Scale **[72]**	MND-SW	Not a measure of Social Inclusion
Ostracism Experience Scale for Adolescents **[73]**	OES-A	Not a measure of Social Inclusion
Friendship Scale **[74]**	N/A	Not a measure of Social Inclusion
Quality of Social-Functioning Scale **[75]**	QOSF	Not a measure of Social Inclusion
Reintegration to Normal Living Index **[76]**	N/A	Not a measure of Social Inclusion
The Participation and Environment measure for Children and Youth **[77]**	PEM-CY	Not a measure of Social Inclusion
Young Children's Participation and Environment Measure **[78, 79]**	YC-PEM	Not a measure of Social Inclusion
The short version of the assessment of Life Habits **[80]**	LIFE-H 3.0	Not a measure of Social Inclusion
The short version of the assessment of Life Habits version 3.1 **[81]**	LIFE-H version 3.1	Not a measure of Social Inclusion
Adolescent Alienation Construct **[82]**	N/A	Not a measure of Social Inclusion
Putnam's Social Capital Index **[83]**	N/A	Not a measure of Social Inclusion
The Annenberg National Health Communication Survey Social Capital Index **[83]**	N/A	Not a measure of Social Inclusion
The Behavioural Risk Factor Surveillance System Measure **[83]**	BRFSS	Not a measure of Social Inclusion
Community Integration Questionnaire **[84, 85]**	N/A	Not a measure of Social Inclusion
Transnational Social Capital Measure **[86]**	NA	Not a measure of Social Inclusion
The Social Acceptance Scale **[87]**	SAS	Not a measure of Social Inclusion
The Lubben social network scale—abbreviated version **[88]**	LNSN-6	Not a measure of Social Inclusion
Pictorial Scale of Perceived Competence and Social Acceptance for Young Children **[89]**	PSPCSC	Not a measure of Social Inclusion
Religious Social Capital measure ^a^ **[90]**	N/A	Not a measure of Social Inclusion
The Global Citizenship Scale **[91]**	N/A	Not a measure of Social Inclusion
The Scale of Participation **[92]**	SCAP	Not a measure of Social Inclusion
The PAR-PRO: a measure of participation **[93]**	NA	Not a measure of Social Inclusion
The Social Capital Questionnaire for Adolescent Students **[94]**	SCQ-AS	Not a measure of Social Inclusion
Social Capital Measure ^a^ **[95]**	NA	Not a measure of Social Inclusion
ICF Measure of Participation and ACTivities Screener part **[96]**	IMPACT-S	Not a measure of Social Inclusion
Clinical Research Trainee Social Capital Scale ^a^ **[97]**	N/A	Not a measure of Social Inclusion
Activity Record **[98]**	AR	Not a measure of Social Inclusion
Temple University Community Participation Measure **[99]**	TUCP	Not a measure of Social Inclusion
The Interpersonal Needs Questionnaire **[100]**	INQ	Not a measure of Social Inclusion
Composite Scale of Social Capital ^a^ **[101]**	N/A	Not a measure of Social Inclusion
Pediatric Community Participation Questionnaire **[102]**	PCPQ	Not a measure of Social Inclusion
The Resource Generator **[103]**	N/A	Not a measure of Social Inclusion
The Resource Generator-UK **[104]**	RG-UK	Not a measure of Social Inclusion
Youth-Adult Partnership Measure **[105]**	Y-AP	Not a measure of Social Inclusion
Perceived Inequality in Childhood Scale **[106]**	PICS	Not a measure of Social Inclusion
Social Attitude Scale **[107]**	N/A	Not a measure of Social Inclusion
Participation objective, Participation subjective measure **[108]**	POPS	Not a measure of Social Inclusion
The Participation Assessment With Recombined Tools-Objective **[109]**	PART-O	Not a measure of Social Inclusion
The Global Social Capital Survey ^a^ **[110]**	N/A	Not a measure of Social Inclusion
The Medical Outcome Study Social Support Survey **[111]**	MOS-SSS	Not a measure of Social Inclusion
Sensory Processing Measure—Preschool **[112]**	SPM-P	Not a measure of Social Inclusion

Notes

^a^ Unofficial title derived from publication content as instruments published without a title.

### Measures of social inclusion

A summary of the studies on the development and validation of the 25 social inclusion measures reviewed is reported in Table 6. One measure was developed using an adolescent sample (12–17 years), with all others using an adult population alone. Of the 25 measures, 10 measures were developed and validated using a sample of adults with severe mental illnesses. Six were developed and validated with community samples; with 3 of these measures using a sample from rural communities or low-income neighbourhoods. Only one measure was developed and validated with both mentally healthy adults and adults with severe mental illnesses (Social and Community Opportunities Profile [SCOPE] [113]). Two measures used samples of adults without a tertiary education, 1 measure used a sample of adults with an intellectual disability and their carers, and 1 measure sampled caregivers of children with chronic illnesses.

**Table 6:** Description of studies for the development and validation of instruments for the assessment of social inclusion.

**Instrument**	**Reference**	**Purpose of study**	**Study population**	**Age (range [R] and/or Mean [M] Standard Deviation [SD])**
**Activity and Participation Questionnaire (APQ)**	Stewart, Sara [114]	Description of development and test-retest reliability of APQ	**N = 123** Adults with diagnosis of schizophrenia or schizoaffective disorder; *Study1*: n = 63; *Study 2*: n = 60	*Total sample*: R = 18-64y; M = NR; SD = NR. *Study 1*: R = NR; M = NR; SD = NR. *Study 2*: R = NR; M = NR; SD = NR
**Australian Community Participation Questionnaire (ACPQ)**	Berry, Rodgers [115]	Development and validation of a community participation questionnaire and an investigation of associations with distress	**N = 963** residents of rural New South Wales, Australia	*Total sample*: R = 19-97y; M = 52.76y; SD = 18.26y
**Bonding Social Capital**	Brisson and Usher [116]	Examination of reliability and validity of the PHDCN’s five items of social cohesion and trust as a bonding social capital scale	**N = 7437** residents of low-income neighbourhoods	*Total sample*: R = NR; M = NR; SD = NR.
**Brief Sense of Community Scale**	Peterson, Speer [117]	To develop and validate original items for inclusion in a new, brief measure of sense of community.	**N = 293** residents of Midwestern United States	*Total sample*: R = NR; M = NR; SD = NR.
**Community Participation Domains Measure (CPDM)**	Chang, Coster [118]	To develop a measure of participation and to assess construct validity with adults with severe mental illnesses.	**N = 235** adults with severe schizophrenia or major affective disorder	*Total sample*: R = NR; M = 47.3y; SD = 9.5y
**Guernsey Community Participation and Leisure Assessment (GCPLA)**	Baker [119]	To develop and assess the validity and reliability of the GCPLA.	**N = 32** *Study 1*: individuals with intellectual disability as respondents n = 12; *Study 2*: individuals with intellectual disability as respondents n = 9; *Study 3*: carers of adults with intellectual disability as respondents n = 12; *Study 4*: carers of adults with intellectual disability as respondents n = 11	*Total sample*: R = NR; M = NR; SD = NR. *Study 1*: R = 20.2–38.7y; M = 27.9y; SD = NR. *Study 2*: R = NR; M = 43.8y; SD = NR. *Study 3*: R = 20.2–38.7y; M = 27.9y; SD = NR. *Study 4*: R = 25-71y; M = 38.6; SD = NR.
**Internet Social Capital Scales (ISCS)**	Williams [120]	To describe the development and validation of the ISCS	**N = 884** adult internet users	*Total sample*: R = 14-68y; M = 27.04y; SD = NR
**Mental health day services and social inclusion questionnaire**	Marino-Francis and Worrall-Davies [121]	The development, validation and testing of reliability of a measure of social inclusion for use in mental health day services.	**N = 78** Adult mental health day services users. *Study 1*: n = 9; *Study 2*: n = 69; *Study 3*: n = 51	*Total sample*: R = NR; M = NR; SD = NR. *Study 1*: R = NR; M = NR; SD = NR. *Study 2*: R = NR; M = NR; SD = NR. *Study 3*: R = NR; M = NR; SD = NR.
**Personal Social Capital scale–English version (PSCS-E)**	Archuleta and Miller [122]	To test the reliability and validity of the PSCS-E	**N = 322** adult students of Mexican descent	*Total sample*: R = 18-65y; M = 31.21y; SD = 11.94y
**Psychological Sense of Community Scale (PSC)**	Jason, Stevens [123]	To construct a new measure of sense of community and evaluate its factor structure and convergent validity	**N = 158** college students of Midwestern United States	*Total sample*: R = NR; M = 20.4y; SD = 3y
**Sense of Community Index (SCI)**	Stevens, Jason [124]	To explore factor structure of the SCI and test whether the measure was predictive of a future behaviour	**N = 662** adult residents of recovery homes. *Sample 1*: n = 316; *Sample 2*: n = 323	*Total sample*: R = NR; M = NR; SD = NR; *Sample 1*: R = NR; M = NR; SD = NR; *Sample 2*: R = NR; M = NR; SD = NR
**Social and Community Opportunities Profile—Long (SCOPE)**	Huxley, Evans [113]	To develop and evaluate the psychometric properties of the long and short forms of the SCOPE	**N = 451** *Sample 1*: mentally healthy adults in the community n = 212; *Sample 2*: adults with common mental disorders n = 40; *Sample 3*: mental health service users n = 43; *Sample 4*: mental health service users n = 40; *Sample 5*: university students n = 119	*Total sample*: R = NR; M = NR; SD = NR; *Sample 1*: R = 16-92y; M = 55y; SD = 21y; *Sample 2*: R = 21-92y; M = 51y; SD = 19y; *Sample 3*: R = 21-67y; M = 49y; SD = 12y; *Sample 4*: R = 22-76y M = 56y; SD = 12y; *Sample 5*: R = NR; M = NR; SD = NR
**Social Capital and Cohesion Scale (SCCS)**	Magson, Craven [125]	To develop the SCCS and test the reliability and validity of the measure	**N = 1371** secondary students	*Total sample*: R = 12-17y; M = NR; SD = NR
**Social Capital Questionnaire (SCQ)**	Onyx and Bullen [126]	Development and validation of the Social Capital Questionnaire	**N = 1211** adults living in rural and urban areas of New South Wales, Australia	*Total sample*: R = 18-65y; M = 38y; SD = 16y
**Social Capital Questionnaire–Revised (SCQ-R)**	O'Brien, Burdsal [127]	Modification and validation of the Social Capital Questionnaire for telephone administration	**N = 496** adults living in an urban community of Midwestern United States	*Total sample*: R = NR; M = NR; SD = NR
**Social Capital Scale**	Looman [128]	To develop and test the validity and reliability of the Social Capital Scale for families of children with special health care needs	**N = 186** caregivers of children aged 4-26y with a chronic health condition. *Study 1*: n = 186; *Study 2*: n = 44	*Total sample*: R = 26-73y; M = 44y; SD = 9.6y
**Social Connectedness Scale**	Lee and Robbins [129]	To report on the development of the SCS, explore factors of the instrument, and test reliability	**N = 616** college students of South-eastern United States. *Sample 1*: n = 313; *Sample*: *2* n = 313; *Sample 3*: n = 18	*Total sample*: R = NR; M = NR; SD = NR; *Sample 1*: R = 17-44y; M = 20.60y; SD = 4.34y; *Sample 2*: R = 17-48y; M = 20.65; SD = 4.61; *Sample 3*: R = 19-48y; M = 23.78; SD = NR
**Social Connectedness Scale–Revised**	Lee, Draper [130]	To revise the SCS, and validate the revisions	**N = 442** college students of North-western United States. *Study 1*: n = 218; *Study 2*: n = 100; *Study 3*: n = 184	*Total sample*: R = NR; M = NR; SD = NR; *Study 1*: R = 17-50y; M = 19.55y; SD = 3.32y; *Study 2*: R = 18-24y; M = 18.89y; SD = 1.15; *Sample 3*: R = 17-23y; M = 18.98y; SD = 1.2y
**Social Inclusion After Transfer (SIT-Instrument)**	de Greef, Segers [131]	To report on the development and validation of the SIT-instrument	**N = 308** “low-educated” adult learners at the completion of an adult education course	*Total sample*: R = NR; M = 57; SD = NR
**Social Inclusion Questionnaire User Experience (SInQUE)**	Mezey, White [132]	To develop and assess the validity of the SInQUE	**N = 66** adults with schizophrenia or schizoaffective disorder living in the community	*Total sample*: R = 23-65y; M = 44y; SD = NR
**Social Inclusion Scale (SIS)**	Secker, Hacking [133]	To develop a measure of social inclusion for use in assessing the outcomes of arts participation for people with mental health needs	**N = 111** adult mental health service users. *Study 1*: n = 23. *Study 2*: n = 88	*Total sample*: R = NR; M = NR; SD = NR. *Study 1*: R = NR; M = NR; SD = NR. *Study 2*: R = NR; M = NR; SD = NR
**Social Inclusion Scale (SIS)**	Wilson and Secker [134]	To assess the validity and reliability of the full and shortened versions of the SIS in a non-clinical population of university students	**N = 103** university students. *Study 1*: n = 103; *Study 2*: n = 95	*Total sample*: R = 18-66y; M = 31.37y; SD = 13.04y. *Study 1*: R = 18-66y; M = 31.37y; SD = 13.04y. *Study 2*: R = NR; M = 31.87y; SD = 13.34y
**Social Participation Questionnaire (SPQ)**	Densley, Davidson [135]	To develop the SPQ by modifying the Social Participation Index and explore its psychometric properties	**N = 789** adults with depressive symptoms	*Total sample*: R = NR; M = NR; SD = NR
**The Inclusion Web**	Hacking and Bates [136]	To describe The Inclusion Web, evaluate the effectiveness of a mental health service, and measure the correlations between scale scores	**N = 149** adult mental health services users	*Total sample*: R = NR; M = NR; SD = NR
**Unnamed**	Lloyd, Waghorn [137]	To assess the internal consistency and the test-retest reliability of a composite measure of social inclusion for people with psychiatric disabilities	**N = 28** adult psychiatric psychosocial rehabilitation service users. *Study 1*: n = 28; *Study 2*: n = 26	*Total sample*: R = NR; M = 37y; SD = 9.1y. *Study 1*: R = NR; M = 37y; SD = 9.1y. *Study 2*: R = NR; M = NR; SD = NR

Table 7 describes the characteristics of the reviewed measures. Of the 25 measures, 19 were published within the last 10 years (since 2005). Regarding the measure type, all used self-report with the exception of the Guernsey Community Participation and Leisure Assessment (GCPLA) which used carer-report if the respondent was unable to answer for themselves [119]. Ten measures collected responses via interviews; seven of which were conducted face-to-face, two of which were conducted over the telephone, and one which was administered via both modalities. Fourteen were self-report questionnaires, 3 of which were administered online, and 11 of which were administered via paper and pen (see Table 7). Response options varied greatly between measures; 16 reported the use of Likert-type scales, and 5 reported differing response types per item. Five of the measures using Likert-type scales reported using a 6-point scale, 7 reported using a 5-point scale, and 3 reported using a 4-point scale. Measures requiring differing response types utilised a combination of ordinal and nominal scales. The Sense of Community Index (SCI) reported the use of a dichotomous (true or false) rating system for its scale [124]. The Inclusion Web utilises a visual “web” in which respondents list people or places under various response categories [136]. Response options for the Activity and Participation Questionnaire (APQ) were not reported [114].

**Table 7:** Characteristics of the instruments for the assessment of social inclusion.

**Instrument**	**Purpose of Instrument**	**Published year**	**Type of measure**	**Subscales/Forms**	**Total number of items**	**Response Options**
APQ **[114]**	A measure of vocational activity and social participation for routine use in community mental health services	2010	Face-to-face interview/telephone interview	*6 “questions”*: (I) Participation in employment; (II) Looking for work (III) Participation in unpaid work; (IV) Participation in study or training; (V) Participation in general community activities; (VI) Readiness to change	31	Not described
ACPQ **[115]**	A measure of community participation	2007	Self-report questionnaire	*14 scales*: Contact with immediate household; Contact with extended family; Contact with friends: Contact with neighbours; Social contact with workmates; Organised community activities; Giving money to charity; Voluntary sector activity; Adult learning; Religious observance; Active interest in current affairs; Expressing opinions publicly; Community activism Political protest	67	*7-point scale*: 1 = never, or almost never to 7 = always, or almost always
Bonding Social Capital **[116]**	A measure of bonding social capital for families living in low-income urban neighbourhoods	2007	Telephone interview	*1 scale*: Bonding social capital	5	*5-point scale*: 1 = low agreement to 5 = high agreement
BSCS **[117]**	A measure of sense of community designed to assess dimensions of needs fulfilment, group membership, influence and emotional connection	2008	Face-to-face interview	*4 scales*: Needs fulfilment; Membership; Influence; Emotional connection	8	*5 point*, *Likert-type scale*: strongly agree to strongly disagree
CPDM **[118]**	A multidimensional measure of participation	2015	Face-to-face interview	*3 scales*: Productivity; Social; Recreation/leisure	25	*Ordinal scale*:1 = enough or more than enough (whether participated or not), 2 = not enough but participated at least one day and 3 = not enough and did not participate; *Nominal scale*: participated at least one day; did not participate
GCPLA **[119]**	To support in the assessment and generation of community participation and leisure needs, and to monitor the outcome of interventions designed to enhance service users’ experience of community and leisure activities	2000	Face-to-face interview (with the individual or carer)	*2 scales*: Frequency of contact; Mode of contact	98	*Frequency items*: 1 = less than every 3 months, 2 = every 3 months or more; 3 = monthly or more frequently, 4 = weekly or more, 5 = daily or more; *Mode items*: 1 = supervised, 2 = accompanied, 3 = alone, 4 = with a peer/group
ISCS **[120]**	To measure the impact of the Internet on social capital	2006	Online survey	*4 scales*: Online bridging; Online bonding; Offline bridging; Offline bonding	40	*5 point Likert scale*: strongly agree to strongly disagree
Mental health day services and social inclusion questionnaire **[121]**	A measure of social inclusion for use in the i3 (mental health) services	2010	Self-report questionnaire	*3 scales*: Relationship with family and friends; Sense of belonging in the community; Participation in society	23	*5 point Likert scale*: 1 = not at all; 2 = occasionally; 3 = sometimes; 4 = often; 5 = all of the time. A 6^th^ option available where relevant: not applicable/not at all
PSCS-E **[122]**	To measure bonding and bridging aspects of social capital	2011	Self-report questionnaire	*2 scales*: Bonding; Bridging	42	*5-point scale*: 1 = a few/none; 5 = a lot/all
PSC **[123]**	To assess sense of community from an ecological perspective	2015	Online survey	*3 scales*: Entity; Membership; Self	24	*6-point scale*: strongly disagree, disagree, slightly disagree, slightly agree, agree, or strongly agree
SCI **[124]**	An instrument for the measurement of sense of community	2011	Survey	*4 scales*: Membership, Influence, Fulfilment of needs; Shared emotional connection	12	*2-point scale*: *true; false*
SCOPE **[113]**	To measure social inclusion for use in the general population, mental health service research, and to evaluate outcomes in in mental health services	2012	Face-to-face interview	*2 forms*: Long; Short *8 scales per form*: leisure and participation, housing and accommodation, safety, work, financial situation, self-reported health, education, family and social relationships	Long: 121 Short: 48	Response types differ per item *Nominal scale*: yes, no; *Nominal scale*: Several scales allowing interviewee to nominate frequency of involvement in activities, reasons for behaviours, types of housing, income, education, or health services accessed; *7-point Likert scale*: 1 = terrible to 7 = delighted; *Short answer*
SCCS **[125]**	To measure social capital	2014	Self-report questionnaire (read aloud to students by a researcher)	*6 scales*: Family Social Capital; Peer Social Capital; Neighbour Social Capital; Institutional Social Capital; School Belonging; School Isolation	29	*5-point Likert scale*: 1 = strongly disagree; 5 = strongly agree
SCQ **[126]**	To measure social capital	2000	Self-report questionnaire	*8 scales*: Participation in the local community; Social Agency, or Proactivity in a Social Context; Feelings of Trust and Safety; Neighbourhood Connections; Family and Friends Connections; Tolerance of Diversity; Value of Life; Work Connections	36	*4-point Likert-type scale*: 1 = no, not much or no, not frequently; 4 = yes, definitely or yes, very frequently
SCQ-R **[127]**	To measure social capital	2004	Telephone interview	See Social Capital Questionnaire	36	See Social Capital Questionnaire
Social Capital Scale **[128]**	To measure investment by families and communities in their relationship with each other, as perceived by the caregiver	2006	Self-report questionnaire	*5 scales*: Community involvement; Sense of belonging; Spiritual community; School connection; Informing/asking	20	*5-point Likert-type scale*: 1 = strongly disagree; 5 = strongly agree
Social Connectedness Scale **[129]**	To measure belongingness by portraying general emotional distance between self and others	1995	Self-report questionnaire	*1 scale*: Social Connectedness Scale	45	*6-point Likert scale*: 1 = strongly agree; 6 = strongly disagree
Social Connectedness Scale–Revised **[130]**	See SCS	2001	Self-report questionnaire	See SCS	20	*6-point Likert scale*: 1 = strongly disagree; 6 = strongly agree
SIT-Instrument **[131]**	To evaluate educational programs for vulnerable adults and their impact on increasing social inclusion	2010	Self-report questionnaire	*6 scales*: Background characteristics; Self-directed learning; Transfer-design; Life-circumstances; Activation and internalization; Participation and connection	147	Response types differ per item *Nominal scale*: yes, no; *Ordinal scale*: totally agree, partly agree, not agree, not disagree, partly disagree, totally disagree; *Ordinal scale*: yes, partly, no; *10 point Likert-scale*: 1 to 10 as self-reflection on statements
SInQUE **[132]**	To measure social inclusion in individuals with severe mental illness	2013	Face-to-face interview	*2 parts*: T1 = the first year prior to first psychiatric admission; T2 = current situation *5 scales per part*: Productivity; Consumption; Access to Services; Political Engagement; Social Integration	*T1*: 28; *T2*: 47	Response types differ per item *Dichotomous scale*: yes/no; *Estimate of frequency*: e.g. “How many neighbours do you know by name?”; *Nominal scale for reasons of non-participation*: lack of money; lack of transport; problems with location; no interest; not available; no time; lack of child care; no one to do it; any other reason
SIS **[134]**	To measure social inclusion when evaluating outcomes of interventions aimed at increasing social inclusion	2009/2015	Self-report questionnaire	*SIS*: *3 scales*: Social Isolation; Social Relations; Social Acceptance. *SIS Short Form*: *1 scale*	*SIS*: 22; *Short Form*: 12	*4 point Likert-type scale*: not at all, not particularly, yes a bit, yes definitely
SPQ **[135]**	To measure social inclusion	2013	Self-report questionnaire	*1 scale*: Social Participation Questionnaire	22	*6-point scale for 18 items*: never, rarely, a few times a year, monthly, a few times a month, once a week or more; *Dichotomous scale for 4 items*: yes, no
The Inclusion Web **[136]**	To provide mental health service users with feedback on social inclusion and to monitor impact of mental health services	2006	Face-to-face interview	*2 scales*: People; Places	16	Respondents list people spoken to and places visited regularly in eight areas of life (Education; Arts and Culture; Faith and Cultural Communities; Services; Employment; Family and Neighbourhood; Volunteering; Sports and Exercise). Responses are tallied for people and places
Unnamed **[137]**	A measure of social inclusion for people with psychiatric disabilities	2008	Face-to-face interview	*5 scales*: Social Valued Role Functioning; Social Support; Stigma Experiences; Integration within the psychosocial rehabilitation setting; Community Integration	59	Response types differ per item *Estimate of frequency*: e.g. Number of days in past week spent providing care for others; *Nominal scale*: yes, no; *Likert scale*: above average to clearly below average; *5-point Likert scale*: never, seldom, sometimes, often, very often; *5-point Likert scale*: always agree, sometimes agree, neutral, sometimes disagree, always disagree.

The domains of social inclusion measured by each instrument are summarised in Table 8. The sub-domains were categorised following a thematic synthesis by two members of the research team of the scales and subscales used by the reviewed measures and, where available, based on the definitions or descriptions of the scales and/or subscales provided in the reviewed studies. Based on the thematic analysis the following sub-domains were identified and subsumed under the most relevant domain: 1) participation (i.e., economic, social and spiritual), 2) connectedness and a sense of belonging (i.e., having a sense of connectedness to family, friends, neighbours, broader community), and 3) citizenship (i.e., political and general community engagement, demonstrating altruism, and having access to community services). Aspects of participation were measured by 19 instruments, 21 instruments evaluated aspects of connectedness and a sense of belonging, and aspects of citizenship were measured by 14 instruments. Ten measures included aspects of all three overarching domains of social inclusion, but no single instrument measured all sub-domains of participation, connectedness and a sense of belonging, and citizenship.

**Table 8:** Domains of social inclusion measured by reviewed instruments.

Domains	Participation			Connectedness and a sense of belonging				Citizenship			
Measures	Economic	Social	Spiritual	Family	Friends	Neighbours	Broader community	Political	Altruism	Community engagement	Access to community services
APQ [114]	X	X									
ACPQ [115]	X		X	X	X	X	X		X		
Bonding Social Capital [116]					X	X	X				
BSCS [117]							X			X	
CPDM [118]	X	X									
GCPLA [119]		X								X	
ISCS [120]		X					X				
Mental health day services and social inclusion questionnaire [121]		X					X				
PSCS-E [122]		X			X	X	X	X	X	X	
PSC [123]		X					X				
SCI [124]						X	X			X	
SCOPE Long [113]	X	X		X	X	X	X				X
SCOPE Short [113]	X	X		X	X	X	X				X
SCCS [125]				X	X	X	X			X	
SCQ [126]		X		X	X		X	X			
SCQ-R [127]		X		X	X		X	X			
Social Capital Scale [128]		X	X	X	X	X	X		X		
Social Connectedness Scale [129]							X				
Social Connectedness Scale–Revised [130]							X				
SIT-Instrument [131]		X		X	X		X				
SInQUE [132]	X				X		X	X			X
SIS [134]		X				X	X			X	X
SPQ [135]		X									
The Inclusion Web [136]		X		X	X		X				
Unnamed [137]	X	X					X			X	

### Psychometric properties

The methodological quality ratings of the studies reviewed are summarised in Table 9. Table 10 summarises the quality of the psychometric properties of the 25 measures based on the quality criteria described by Terwee, Bot [29] and Schellingerhout, Verhagen [30] (see Table 3). Table 11 provides an overall psychometric quality rating for each psychometric property using the criteria of Schellingerhout, Verhagen [30]. A description of the criteria used to rate psychometric quality is provided in the notes section for Table 10. As described by Schellingerhout, Verhagen [30], the overall level of psychometric quality (Table 11) is derived by integrating the ratings of 1) the methodological quality of the studies using the COSMIN checklist (Table 9); and 2) the quality criteria for the psychometric properties of assessments (Table 10).

**Table 9:** Overview of the psychometric measurement properties of social inclusion instruments.

Instrument	Authors	Year	Internal consistency	Reliability	Measurement error	Content validity	Structural validity	Hypotheses testing
**APQ**	Stewart, Sara [114]	2010	NR	Excellent (81.3, 85.4)	NR	Excellent (95.0)	NR	NR
**ACPQ**	Berry, Rodgers [115]	2007	Excellent (100.0)	NR	NR	Excellent (95.0)	Excellent (83.3)	Excellent (92.5)
**Bonding Social Capital**	Brisson and Usher [116]	2007	Excellent (84.4)	NR	NR	NR	Good (62.5)	NR
**BSCS**	Peterson, Speer [117]	2008	NR	NR	NR	NR	Good (62.5)	Excellent (80.0, 80.0, 75.0, 77.5)
**CPDM**	Chang, Coster [118]	2015	Excellent (78.1)	NR	NR	Excellent (95.0)	Good (67.9)	Excellent (75.0, 75.0, 78.1)
**GCPLA**	Baker [119]	2000	Good (59.4)	Good (75.0, 68.2, 68.2)	NR	Excellent (95.0)	NR	Good (65.6, 62.5, 67.5, 67.5)
**ISCS**	Williams [120]	2006	Excellent (93.8)	NR	NR	NR	Good (75.0)	Good (75.0)
**Mental health day services and social inclusion questionnaire**	Marino-Francis and Worrall-Davies [121]	2010	Good (68.8)	Good (75.0)	NR	Good (60.0)	Good (66.7)	NR
**PSCS-E**	Archuleta and Miller [122]	2011	Excellent (100.0)	NR	Fair (47.7)	NR	Excellent (85.7)	Excellent (92.5, 90.0, 93.8)
**PSC**	Jason, Steven[123]	2015	Excellent (83.3)	NR	NR	NR	Good (67.9)	Good (75.0, 72.5, 67.5, 70.0)
**SCI**	Stevens, Jason [124]	2011	Excellent (100)	NR	NR	NR	Excellent (83.3)	NR
**SCOPE Long**	Huxley, Evans [113]	2012	Good (65.6)	NR	NR	Fair (45.0)	NR	Good (71.9, 71.9, 71.9, 75.0, 75.0)
**SCOPE Short**	Huxley, Evans [113]	2012	Good (71.9)	Excellent (86.4)	NR	Good (70.0)	Fair (50.0)	Good (75.0, 75.0)
**SCCS**	Magson, Craven [125]	2014	Excellent (87.5)	NR	NR	Excellent (80.0)	Good (62.5)	Good (75.0)
**SCQ**	Onyx and Bullen [126]	2000	NR	NR	NR	Good (65.0)	Excellent (83.3)	Excellent (87.5)
**SCQ-R**	O'Brien, Burdsal [127]	2004	NR	NR	NR	NR	Good (75.0)	Excellent (78.1)
**Social Capital Scale**	Looman [128]	2006	Good (69.4)	Good (75.0)	NR	NR	Good (62.5)	Good (68.8, 68.8, 71.9)
**Social Connectedness Scale**	Lee and Robbins [129]	1995	Excellent (86.1)	Good (72.7)	NR	Excellent (95.0)	Good (62.5)	NR
**Social Connectedness Scale–Revised**	Lee, Draper [130]	2001	Excellent (86.1)	NR	NR	Excellent (80.0)	Good (62.5)	Excellent (85.0, 85.0, 85.0, 85.0)
**SIT-Instrument**	de Greef, Segers [131]	2010	Excellent (77.8)	NR	NR	Excellent (100)	Fair (50.0)	NR
**SInQUE**	Mezey, White [132]	2013	NR	NR	NR	NR	NR	Good (72.5)
**SIS**	Wilson and Secker [134]	2015	Good (68.8)	Good (55.0)	NR	NR	NR	Excellent (80.0)
**SIS**	Secker, Hacking [133]	2009	Good (75.0)	NR	NR	NR	NR	Good (67.5)
**SPQ**	Densley, Davidson [135]	2013	Excellent (84.4)	NR	NR	NR	Good (67.9)	NR
**The Inclusion Web**	Hacking and Bates [136]		NR	NR	NR	NR	NR	Good (60.0)
**Unnamed**	Lloyd, Waghorn [137]	2008	Fair (37.5)	Good (68.2)	NR	NR	NR	NR

*Notes*: The measurement properties of each instrument were evaluated according to the COSMIN rating. A four-point rating scale was used (1 = Poor, 2 = Fair, 3 = Good, 4 = Excellent) and the outcome presented as percentage of rating (Poor = 0.0%-25.0%, Fair = 25.1% -50.0%, Good = 50.1%-75.0%, Excellent = 75.1%-100.0%); NR = Not reported; NA = Not applicable; Measurement properties of criterion validity and cross-cultural validity were not within the scope of this review.

**Table 10:** Quality of psychometric properties based on the criteria by Terwee, Bot [29] and Schellingerhout, Verhagen [30].

**Instrument**	**Reference**	**Internal consistency**	**Reliability**	**Measurement error**	**Content validity**	**Structural validity**	**Hypotheses testing**
**APQ**	Stewart, Sara [114]	NR	-	NR	+	NR	NR
**ACPQ**	Berry, Rodgers [115]	?	NR	NR	?	+	-
**Bonding Social Capital**	Brisson and Usher [116]	+	NR	NR	NR	?	NR
**BSCS**	Peterson, Speer [117]	NR	NR	NR	NR	?	-
**CPDM**	Chang, Coster [118]	?	NR	NR	-	?	-
**GCPLA**	Baker [119]	?	?	NR	+	NR	?
**ISCS**	Williams (120)	?	NR	NR	NR	+	-
**Mental health day services and social inclusion questionnaire**	Marino-Francis and Worrall-Davies [121]	?	-	NR	+	+	NR
**PSCS-E**	Archuleta and Miller [122]	+	NR	?	NR	+	-
**PSC**	Jason, Stevens [123]	+	NR	NR	NR	?	-
**SCI**	Stevens, Jason [124]	-	NR	NR	NR	?	NR
**SCOPE Long**	Huxley, Evans [113]	?	NR	NR	-	NR	±
**SCOPE Short**	Huxley, Evans [113]	?	+	NR	+	?	+
**SCCS**	Magson, Craven [125]	+	NR	NR	?	?	?
**SCQ**	Onyx and Bullen [126]	NR	NR	NR	?	+	?
**SCQ-R**	O'Brien, Burdsal [127]	NR	NR	NR	NR	?	?
**Social Capital Scale**	Looman [128]	?	-	NR	NR	+	+
**Social Connectedness Scale**	Lee and Robbins [129]	+	?	NR	+	+	NR
**Social Connectedness Scale–Revised**	Lee, Draper [130]	+	NR	NR	-	+	-
**SIT-Instrument**	de Greef, Segers [131]	?	NR	NR	+	?	NR
**SInQUE**	Mezey, White [132]	NR	NR	NR	NR	NR	-
**SIS**	Wilson and Secker [134]	?	?	NR	NR	NR	+
**SIS**	Secker, Hacking [133]	+	NR	NR	NR	NR	+
**SPQ**	Densley, Davidson [135]	?	NR	NR	NR	?	NR
**The Inclusion Web**	Hacking and Bates [136]	NR	NR	NR	NR	NR	?
**Unnamed**	Lloyd, Waghorn [137]	?	?	NR	NR	NR	NR

*Notes*: The quality of the psychometric properties of each instrument were evaluated according to the criteria set out by Terwee, Bot [29] and Schellingerhout, Verhagen [30]. + = positive rating;? = Indeterminate rating;— = negative rating; ± = conflicting data; NR = Not reported; NE = Not evaluated; Measurement properties of criterion validity and cross-cultural validity were not within the scope of this review.

**Table 11:** Overall quality score of assessments for each psychometric property based on levels of evidence by Schellingerhout, Verhagen [30].

**Instrument**	**Internal consistency**	**Reliability**	**Measurement error**	**Content validity**	**Structural validity**	**Hypotheses testing**
**APQ [114]**	NR	Strong (negative result)	NR	Strong (positive result)	NR	NR
**ACPQ [115]**	Indeterminate	NR	NR	Indeterminate	Strong (positive result)	Strong (negative result)
**Bonding Social Capital [116]**	Strong (positive result)	NR	NR	NR	Indeterminate	NR
**BSCS [117]**	NR	NR	NR	NR	Indeterminate	Strong (negative result)
**CPDM [118]**	Indeterminate	NR	NR	Strong (negative result)	Indeterminate	Strong (negative result)
**GCPLA [119]**	Indeterminate	Indeterminate	NR	Strong (positive result)	NR	Indeterminate
**ISCS [120]**	Indeterminate	NR	NR	NR	Moderate (positive result)	Moderate (negative result)
**Mental health day services and social inclusion questionnaire [121]**	Indeterminate	Moderate (negative result)	NR	Moderate (positive result)	Moderate (positive result)	NR
**PSCS-E [122]**	Strong (positive result)	NR	Indeterminate	NR	Strong (positive result)	Strong (negative result)
**PSC [123]**	Strong (positive result)	NR	NR	NR	Indeterminate	Strong (negative result)
**SCI [124]**	Strong (negative result)	NR	NR	NR	Indeterminate	NR
**SCOPE Long [113]**	Indeterminate	NR	NR	Limited (negative result)	NR	Conflicting
**SCOPE Short [113]**	Indeterminate	Strong (positive result)	NR	Moderate (positive result)	Indeterminate	Moderate (positive result)
**SCCS [125]**	Strong (positive result)	NR	NR	Indeterminate	Indeterminate	Indeterminate
**SCQ [126]**	NR	NR	NR	Indeterminate	Strong (positive result)	Indeterminate
**SCQ-R [127]**	NR	NR	NR	NR	Indeterminate	Indeterminate
**Social Capital Scale [128]**	Indeterminate	Moderate (negative result)	NR	NR	Moderate (positive result)	Strong (positive result)
**Social Connectedness Scale [129]**	Strong (positive result)	Indeterminate	NR	Strong (positive result)	Strong (positive result)	NR
**Social Connectedness Scale–Revised [130]**	Strong (positive result)	NR	NR	Strong (negative result)	Moderate (positive result)	Strong (negative result)
**SIT-Instrument [131]**	Indeterminate	NR	NR	Strong (positive result)	Indeterminate	NR
**SInQUE [132]**	NR	NR	NR	NR	NR	Moderate (negative result)
**SIS [134]**	Moderate (positive result)	Indeterminate	NR	NR	NR	Strong (positive result)
**SPQ [135]**	Indeterminate	NR	NR	NR	Indeterminate	NR
**The Inclusion Web [136]**	NR	NR	NR	NR	NR	Indeterminate
**Unnamed [137]**	Indeterminate	Indeterminate	NR	NR	NR	NR

*Notes*: Levels of Evidence: Strong evidence positive/negative result = Consistent findings in multiple studies of good methodological quality OR in one study of excellent methodological quality; Moderate evidence positive/negative result = Consistent findings in multiples studies of fair methodological quality OR in one study of good methodological quality; Limited evidence positive/negative = One study of fair methodological quality; Conflicting evidence = Conflicting findings; Not Evaluated = studies of poor methodological quality according to COSMIN excluded from further analyses; Indeterminate = Studies with Indeterminate measurement property rating; NR = Not reported. Measurement properties of criterion validity and cross-cultural validity were not within the scope of this review.

## Discussion

The purpose of this systematic review was to identify and evaluate the extent to which contemporary measures of social inclusion evaluate the construct in any population group, and the quality of their psychometric properties. The measurement of social inclusion is important to policy makers in health and social services as it can bring together a combination of economic, social, geographical and individual factors; the combination of which are increasingly being understood to influence health and social outcomes of populations. The systematic review of social inclusion measures provides a comprehensive summary of the quality of the psychometric properties of these measures.

### Findings on psychometric properties

The systematic review identified a total of 25 measures published across 25 papers and 1 manual. For 24 measures, only single studies were identified reporting on one or more of the psychometric properties within the scope of this review. Only the SIS had two psychometric studies. Most studies only addressed a few of the six measurement properties evaluated within this review (average 3; range 1–5). Furthermore, when determining the overall quality score per psychometric property per measure, 45% of the overall ratings was classified as indeterminate. Consequently, the reporting of psychometric properties of social inclusion measures within the literature paints an incomplete picture. The lack of psychometric data in the literature is worrying. Whilst missing data do not necessarily indicate poor psychometric quality, without this knowledge clinicians and researchers are selecting measures based on incomplete psychometric evidence. Missing data on reliability, validity and responsiveness of measures, have an impact on the generalisability and interpretation of results.

Evaluation of the reliability (internal consistency, test-retest, interrater or measurement error) was conducted on a majority of reviewed measures (20 of 26). Internal consistency was the most frequently reported psychometric domain and was evaluated with strong methodological quality producing Cronbach’s alphas in the acceptable range in six instruments. In addition, the SCOPE-Short produced strong evidence for test-retest reliability. Issues with methodological quality were usually the reason for “indeterminate” results in the final overall quality scores for internal consistency, reliability and measurement error. In evaluations of internal consistency, most studies failed to collect an adequate sample size for the number of items in the instrument of focus. This may have been because of the specific population groups for which the instruments were validated. Problems with the recruitment of hard to reach populations (e.g., adults with mental illnesses, rural communities, those from low socioeconomic areas) may have reduced the study sample sizes, and these instruments require further validation with larger sample sizes so that conclusions about their psychometric properties can be drawn. Notably, when an adequate sample size was collected, internal consistency results were usually positive. Further methodological problems were evident in most evaluations of test-retest reliability, with researchers opting to report Pearson’s or Spearman’s correlations rather than Kappa or ICCs.

All but one instrument underwent an evaluation of at least one aspect of validity (i.e., content validity, structural validity and/or hypotheses testing). Results for all instruments were mixed, with many finding positive results in one aspect of validity and negative or indeterminate results in another. Inadequate reporting led to “indeterminate” results for the overall quality assessment of structural and content validity. Specifically, descriptions of measurement aims, target populations, concepts measured and means of item selection estimates were unclear or absent from studies reporting on content validity. Additionally, estimates of variance were not reported in some studies of structural validity. However, when adequate reporting was detected, overall quality scores for content validity and structural validity were usually positive. Most results for hypotheses testing were deemed indeterminate or negative. Indeterminate results were due to inadequate sample sizes and when studies utilised adequate sample sizes, ratings were often negative due to weak (*r* < 0.5) and/or statistically insignificant correlations. Criterion validity could not be assessed due to the absence of a “gold standard” measure for social inclusion, and cross-cultural validity was outside the scope of this review.

When considering those measures that showed no negative psychometric evidence (13 measures), the Social Connectedness Scale and the SCOPE Short seem to be the most promising measures. For the Social Connectedness Scale, strong positive psychometric evidence was found on three properties (internal consistency, content validity and structural validity) and indeterminate evidence on a fourth property (reliability). For the SCOPE-Short, strong positive evidence was found for reliability, moderate positive evidence for content validity and hypotheses testing, and indeterminate ratings for internal consistency and structural validity. Next, the SIS showed strong and moderate positive evidence on two properties: hypotheses testing and internal consistency, respectively. Data on reliability scored indeterminate. The other ten measures without negative evidence ratings, showed either positive evidence on single psychometric properties (5 measures) or indeterminate ratings (4 measures) only, resulting in very incomplete psychometric overviews for these measures. Four measures showed only negative psychometric evidence (BSCS, CPDM, SCI and SinQUE) in addition to indeterminate ratings. Finally, eight measures showed a combination of positive and negative evidence for at least two psychometric properties. When considering the overall psychometric quality scores for all 25 measures, many data proved missing or indeterminate and indicated an urgent need for further research to determine the psychometric properties of these measures. Further, the use in policy evaluation and clinical practice of measures having poor psychometric properties should not be supported.

Overall, the results demonstrate that the current suite of available instruments for the measurement of social inclusion is promising, but requires further refinement. There is a need for researchers to utilise more robust methodology when evaluating psychometrics, particularly in relation to the collection of adequately sized samples and the selection of statistical tests. While no instrument received a “poor” rating for methodological quality, flaws in methodology reduced the ability to draw conclusions about results in many studies. There is also a need for more complete reporting of instrument purpose, concepts assessed, target populations, and selection of items. Without this knowledge, there is a risk of clinicians, researchers and policy developers making inappropriate instrument selections.

The findings of the review also support the need for further consideration of instrument design when attempting to measure social inclusion. All identified instruments were self-report measures. While there are a number of advantages to using self-report measures, a disadvantage of self-report methodology is the potential for inaccurate reporting by the respondent [138]. Similarly, some of the measures used Likert scales in combination with dichotomous and nominal scales. Deciding on a scale and response format to use is not simple and requires attention to the meaning of the terms and words as well as the context [139]. Other design considerations emerged related to the fact that all but one of the identified measures (SCCS) were developed and validated with adults only. Moreover, the most frequently sampled population for the development and validation of the social inclusion measures was adults diagnosed with mental health problems. Further validation of instruments for the general populations, as well as populations at risk of social exclusion would allow researchers and policy makers to evaluate the impact of social policies and specific interventions for population subgroups as well as the broader population.

### Social inclusion theory and measurement

The systematic review utilised social inclusion theory to inform a deductive thematic analysis of the findings [140]. The three domains of social inclusion (i.e., participation, connectedness and a sense of belonging, and citizenship) were used to analyse the reviewed instruments in relation to how comprehensively they assess the construct of social inclusion. The domain of participation includes the sub-domains of economic, social and spiritual participation. The domain of connectedness included four sub-domains: family, friends, neighbours, and broader community. Finally, the domain of citizenship comprised of four sub-domains: political, altruism, community engagement and access to community services. No single measure captured the complexities of social inclusion represented by these domains, and as such we have identified gaps in measuring social inclusion from a theoretical perspective.

*Participation* as a domain of social inclusion has previously been identified as an important predictor of social inclusion [141]. Often, vulnerable populations are left marginalised and at risk of reduced opportunities to participate in society [118]. The three sub-domains of economic, social and spiritual participation were identified as being consistent with how individuals contribute to and participate in their community.

The sub-domain of *economic participation* included employment, self-employment enterprise development, education and training [142]. Seven out of the 25 identified measures included the sub-domain of economic participation, but definitions of work and paid employment varied between measures. Some measures focused primarily on paid employment as a gateway to participation in society, however employment is not a sole guarantor of social inclusion [143]. As such, measures of social inclusion require broader consideration and examination of the concept of work, employment and education. From this perspective, facilitating participation to enhance social inclusion requires more than enabling people to enter paid employment. A broad perspective of a person’s means of contributing, participating and belonging to society is required [99].

Social roles are thought to be a nuanced aspect of participation and more than simple engagement in daily activities [42]. At a societal level, The World Health Organization interprets *social participation* within a number of different forms, including empowering communities to retain ultimate control over the key decisions that affect their wellbeing [144]. At the level of the individual, social participation includes participation in formal community organisations, informal community networks and activities, volunteer work, and care of family (including children and elderly) [142]. Kawachi, Kennedy [145] indicate low social participation may be a pathway associated with deprivation and poor health, and reduced social support and anchorage are often negatively associated with poor mental health outcomes [146]. Seventeen out of the 25 identified measures included the sub-domain social participation.

The remaining sub-domain of participation, *spirituality*, relates to participation in groups and activities with others who have similar beliefs and a common way of worship. A number of studies have noted that amongst other benefits, education and awareness around religious diversity and spirituality has an important role in advancing social inclusion [147]. Yet, only two of the 25 identified measures incorporated questions related to sub-domain of spirituality, making it an under-recognised aspect in the measurement of social inclusion.

The domain of *connectedness and a sense of belonging* relates to relationships within societal groups and associated feelings of emotional attachment [17]. Connectedness identifies social norms within a group (i.e., family or friendships) that may provide strong motivation to remain connected [148, 149]. Belongingness, a fundamental human need [150], adds an emotional aspect to the domain, as it is possible to be connected but not emotionally attach. As such, to facilitate true social inclusion a person needs to be both connected and have a sense of belonging. Becoming involved in community groups or organisations is one way of increasing a sense of connection and belongingness in a complex and fragmented society [151], however complete interpersonal integration means having a diversity in social networks (e.g., family, friends, neighbours, community groups) to provide care and companionship and moral support [113]. This systematic review identified nine measures that included the sub-domain of family, 12 that included the sub-domain of friends, and nine included the sub-domain of neighbours. With twenty-one out of the 25 identified measures having included the sub-domain of broader community connectedness and sense of belonging, it was the most common sub-domain captured within the measures of social inclusion.

In contrast to the connectedness domain, the domain of *citizenship* considers social inclusion as more than just participation and belonging within family, friendship and other social networks. Citizenship implies membership in a community with associated rights and obligations, and the ‘extent’ of citizenship is determined by the rules and norms of inclusion and exclusion that a society develops to define the boundaries of membership [152]. As per the sub-domains of citizenship adopted for this review, an individual can exercise citizenship through community engagement, community service access, political activism, and acts of altruism.

Social inclusion requires opportunities for *community engagement*, which in turn creates opportunities to reduce health inequities and increase positive mental and physical health outcomes [153–156]. The sub-domain of *access to community services* was also included as a sub-domain in this review, because accessing services is very different from engaging in the community. Community engagement was the most frequently measured sub-domain of citizenship, found in 11 of the 25 measures reviewed. Accessing community services was again an under-evaluated concept, appearing in four of the 25 measures.

The World Summit for Social Development [157] considers an inclusive society as one in which every individual has an active role in meeting their own rights and responsibilities. This highlights the importance of *political action* within the construct of social inclusion. Political action provides an avenue for individuals to influence their rights and responsibilities, and this is realised by accessing a sense of trust gained from reciprocal contribution to a network [158]. The notion of being able to “have a say” bestows a sense of empowerment upon the individual, and to be included in society there must be opportunities to have a political voice and take political action [159]. *Altruism* has also been shown to influence behaviours toward an inclusive community [160], and Cobigo, Ouellette-Kuntz [141] propose that the definition and value of social capital must also include *altruism*. Four of the 25 measures reviewed included the sub-domain of political action and three included the sub-domain of altruism, highlighting these as under-evaluated domains within the construct of social inclusion.

When we integrate the findings from the psychometric qualities of the identified measures with how well the measures cover the construct of social inclusion from a theoretical perspective, the SCOPE Short [113] has shown itself to be the most promising measure of social inclusion (covering 7 out of 11 sub-domains), followed by the SIS [134] (covering 5 out of 11 subdomains). While the quality of the psychometric properties of the Social Connectedness Scale [129] shows promise, it is narrow in its measurement of the construct of social inclusion. Overall the findings highlight the need for more research to fully capture the complex construct of social inclusion and to validate the measures using sound psychometric methodologies.

## Conclusion

This systematic review reported evidence of the quality of psychometric properties of the 25 instruments used to measure social inclusion with any population. The COSMIN taxonomy, [22] was used to rate the reliability and validity information reported about the instruments. No single measure of social inclusion was found to demonstrate a consistent level of psychometric evidence across the six psychometric properties appraised. The research findings indicate there is then a need for a “gold standard” measure of social inclusion that utilises a more vigorous methodological design, including using adequate sample sizes and appropriate statistical analyses. Furthermore, the breadth of the definition of social inclusion highlights the necessity for having an expansive measure to fully capture all the nuances of the highly complex construct. None of the identified measures completely capture all aspects associated with social inclusion across the domains of participation, connectedness and a sense of belonging, and citizenship. The SCOPE-Short was the measure with the best evidence of sound psychometric properties and covering the breadth of the construct of social inclusion. In conclusion, a broad-based measure of social inclusion can offer policy makers with the opportunity to develop an evidence base that can be used to underpin the development of health and social policies and evaluate their impact following implementation.

## Supporting information

S1 Table PRISMA checklist. (DOCX)
